# Prevalence of Prediabetes and Type 2 Diabetes Mellitus in Football Players: A Novel Multi Football Clubs Cross Sectional Study

**DOI:** 10.3390/ijerph18041763

**Published:** 2021-02-11

**Authors:** Sultan Ayoub Meo, Abdulelah Adnan Abukhalaf, Ali Abdullah Alomar, Omar Mohammed Alessa, Omar Yassin Sumaya, Anusha Sultan Meo

**Affiliations:** 1 Department of Physiology, College of Medicine, King Saud University, Riyadh 11461, Saudi Arabia; Abdulelahabukhalaf@gmail.com (A.A.A.); AliAlomarMD@gmail.com (A.A.A.); omar.m.alessa@gmail.com (O.M.A.); iomar.y.s96@gmail.com (O.Y.S.); 2 Army Medical College, National University of Medical Sciences (NUMS), Rawalpindi 051, Pakistan; anushasultan@hotmail.co.uk

**Keywords:** football, sports, prevalence, diabetes mellitus

## Abstract

Sports offer great benefits, improving health and reducing the risk of illnesses. This study’s aim was to investigate the prevalence of prediabetes and type 2 diabetes mellitus in football players compared to population based non-elite athlete control subjects. Initially 1100 male volunteers, (550) football players, and (550) population based non-elite athlete control subjects were interviewed. After socio-demographic and medical history analysis, 756 (378) nonsmoker male football players and (378) nonsmoker male control subjects were recruited. The control subjects were not involved in regular sports activities such as football, volleyball, badminton, cricket, hockey, and swimming. Participants with a known history of anemia, blood diseases, diabetes mellitus, and malignancy were excluded from the study. The mean age of football players was 31.80 ± 5.46 years, Body Mass Index (BMI) was 26.40 ± 2.08 (kg/m^2^), and the mean age of control subjects was 32.32 ± 4.37 years, and BMI was 26.66 ± 1.87 (kg/m^2^). The selected football players have been playing football for about 2 h a day, 3 days per week, and so the total mean duration of playing football was 1.08 years. American Diabetes Association (ADA) based criteria on Glycated Hemoglobin (HbA1c) was used to investigate prediabetes and type 2 diabetes mellitus. In football players the prevalence of prediabetes was 30 (7.93%) and type 2 diabetes mellitus (T2DM) was 6 (1.59%) compared to population based matched non-elite athlete control subjects where the prediabetes was 71 (18.78%) and T2DM was 89 (23.54%) (*p* = 0.001). Among football players there was a 7-fold decrease in T2DM compared to control subjects. Football recreational activities markedly reduce the prevalence of prediabetes and T2DM. The study findings demonstrate the benefits of football and other such sport activities and emphasize the urgent need for promoting football based physical activities as a physiological preventive strategy against the globally growing diabetes epidemic.

## 1. Introduction

Diabetes mellitus (DM) is a global health challenge associated with substantial morbidity, mortality, and economic burden [1]. In spite of advancements in biomedical sciences, DM is an incurable life-long disease [2]. The recent global prevalence of DM is 463 million; 374 million people are suffering from impaired glucose tolerance whereas 232 million people are unaware from the fact that they are suffering from the disease. Diabetes caused 4.2 million deaths in the year 2019, 11,666 people per day, and 8.10 people per minute. Moreover, the world health expenditure on diabetes is 760 billion $ [3]. 

Currently, diabetes mellitus has a high priority rank on the international health agenda due to being a global pandemic and a deathtrap to human health and worldwide economies [4]. Worldwide, many countries have implemented policies to arbitrate on risk factors, such as “lifestyle, smoking, diet, physical activity” to minimize the prevalence of diabetes mellitus [5]. Lack of regular physical activities, unhealthy diet, and similar lifestyles account for increasing obesity and diabetes mellitus [6]. 

Football-based sport has been acknowledged as a potential health promotion strategy to reduce the sedentary behavior. Football improves endurance capacity and has a positive influence on cardiovascular and metabolic health [7]. Football based physiological involvements achieve the primary preventive effects and improvements in human health [8]. In recent years, the evidence for the health benefits of football sport showed that it improves aerobic fitness, muscular performance, metabolic and cardiovascular function, and reduces adiposity [9]. However, literature is extremely lacking to establish an association between playing football and prevalence of prediabetes and type 2 diabetes mellitus (T2DM). This study’s aim was to investigate the prevalence of prediabetes and T2DM in football players compared to population based non-elite athlete matched control subjects. 

## 2. Subjects and Methods

### 2.1. Study Participants

**Figure 1:**
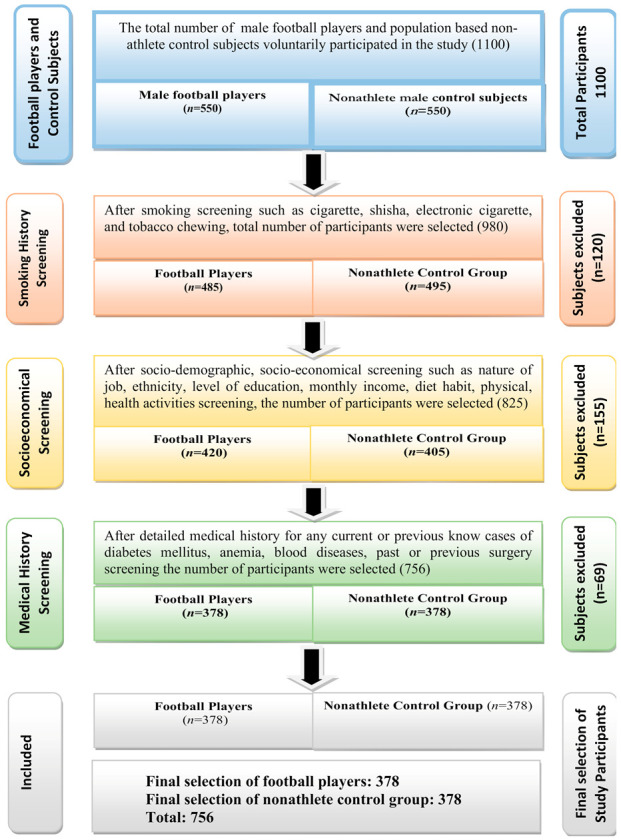
Flow diagram of the selection of football players and population based nonathlete control subjects.

In this study, various schools, colleges, universities, small and large-scale football sport grounds were randomly visited and information about football players was gathered. Initially 1100 volunteer males, (550) football players, and (550) population based non-elite athlete control subjects were interviewed. After socio-demographic, medical history and examination, a total of 756 (378) nonsmoker male football players and (378) nonsmoker control subjects were recruited (Figure 1). Power analysis was used to calculate the sample size. The mean age of football players was 31.80 ± 5.46 years, weight 77.81 ± 6.88 kg, and Body Mass Index (BMI) 26.40 ± 2.08 (kg/m^2^). The selected football players have been playing football for about 2 h a day, 3 days per week; the total mean duration of playing football was 12.98 ± 0.47 months (Table 1). It was ensured that these players were involved in football sport only and no other sports allied activities such as volleyball, badminton, cricket, hockey, swimming etc. Moreover, these football players were not involved in working exposure to any industries such as cement, coal, cotton, oil, and flour factories as these industries generate pollution, and pollution increases the prevalence of diabetes mellitus [2,10,11].

Similarly, for control group, various schools, colleges, universities were randomly visited and initially, 550 population based non-elite athlete control subjects were interviewed. After socio-demographic and medical history examination, 378 control subjects were selected from schools and universities’ clerical staff, technicians, and research assistants. The mean age for the non-elite athlete control subjects was 32.32 ± 4.37 years, weight 76.85 ± 6.99 kg and Body Mass Index 26.66 ± 1.87 (kg/m^2^). It was warranted that these control subjects were not involved in regular sports activities such as football, volleyball, badminton, cricket, hockey, swimming, etc. Moreover, these control subjects were not involved in working exposure to any industries such as cement, coal, cotton, oil, and flour factories as these industries generate pollution, and pollution increases the prevalence of diabetes mellitus [2,10,11]. A verbal consent was obtained from the participants who had voluntarily registered in the research project. 

### 2.2. Clinical History and Socio Demographic Characteristics

Three co-investigators interviewed 550 volunteer male football players and a detailed sociodemographic and medical history was obtained. The information about “age, gender, height, weight, BMI, duration of playing football, demographic characteristics, lifestyle, dietary habit, physical activities and other health-related information” were collected by the use of a questionnaire. Moreover, the “socio-demographic characteristics including residential address, living conditions, education level, marital status, monthly income, lifestyle information, and smoking” were recorded. Other health-related evidence including family history of diabetes mellitus was also taken. Both groups were matched for, “age, weight, BMI, socioeconomical, and dietary habits”. After demographic, medical history and examination, a final total of 756, (378) nonsmoker football players, and (378) nonsmoker control subjects were recruited (Figure 1).

### 2.3. Exclusion Criteria 

Participants with a “known history of anemia, blood diseases, blood transfusion, asthma, diabetes mellitus, and malignancy were excluded from the study”. Subjects who smoke traditional or electronic cigarette or shisha were also excluded [12]. It was ensured that the football players were only playing football and control group participants were population based non-elite athlete subjects. The participants with a current or previous history of an employment in any industrial plant which produces dust or fumes such as plastic, cement, coal, cotton, and flour factories were also not included in the study [10,11] (Figure 1).

### 2.4. Measurements of Glycated Hemoglobin (HbA1c)

After detailed interview, the participants were allocated an identification number, and a para-medical staff measured the HbA1c, by using the “Clover A1c system (Inforpia, Kyunggi, Korea), an automated boronate affinity assay for the determination of the percentage of HbA1c % in the whole body’s blood” [13]. American Diabetes Association (ADA) [14] based criteria on glycated hemoglobin (HbA1c) was used to diagnose the diabetes mellitus. Subjects with “HbA1c less than 5.7% were considered as non-diabetics; HbA1c 5.7–6.4% as prediabetics; and subjects with HbA1c more than 6.4% were considered diabetics” [14]. HbA1c is a reliable indicator of glycemic measurements for the diagnosis of diabetes mellitus [14,15]. 

### 2.5. Ethics Statement

This study was executed in harmony with the “Declaration of Helsinki”, and the protocol was approved by the “Ethics Committee, College of Medicine Research Centre, King Saud University (E-19-4494)”.

### 2.6. Statistical Analysis

The continuous variables were expressed as the Mean ± Standard Deviation and descriptive data were expressed as frequency (%). The frequencies and percentages for prevalence of prediabetes and Type 2 Diabetes Mellitus, their association with social-demographics data and duration of playing football was calculated by using chi-square tests of independence. Pearson correlation coefficient regression model was used to identify the independent risk. The level of significance was presumed at *p* < 0.05. 

## 3. Results

The anthropometric characteristics of the football plyers and control subjects are presented in Table 1. The mean age of football payers was 31.80 ± 5.46 years, weight 77.81 ± 6.88 kg, and Body Mass Index 26.40 ± 2.08 (kg/m^2^); and the mean age of control subjects was 32.32 ± 4.37 years, weight 76.85 ± 6.99 kg, and Body Mass Index 26.66 ± 1.87 (kg/m^2^) (Table 1). The selected football players have been playing football for about 2 h a day, 3 days per week, the mean duration of playing football was 12.98 ± 0.47 months. Both of the groups were matched for age, weight, BMI (Table 1), diet habit, socioeconomic, and educational levels. 

**Figure 2:**
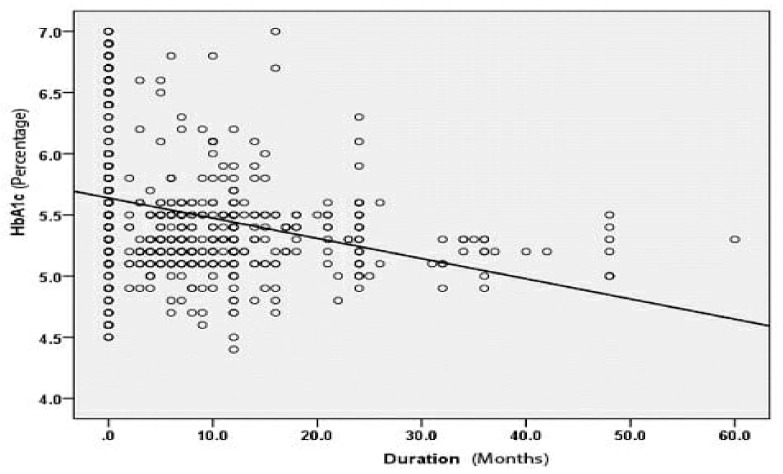
Correlation between duration of playing football and HbA1c.

In football players the prevalence of prediabetes was 30 (7.93%) and type 2 diabetes mellitus (T2DM) was 6 (1.59%) compared to population based matched non-elite athlete control subjects where the prediabetes was 71 (18.78%) and T2DM was 89 (23.54%) (*p* = 0.001) (Table 2). The prevalence of prediabetes and T2DM among the football players was significantly decreased with duration of playing football (Figure 2). Among football players there was a 7 fold decrease in T2DM compared to control subjects (Table 2).

The correlations between age, BMI, and duration of playing football and level of HbA1c showed that there was a significant association between the duration of playing football and deceased level of HBA1c. However, this association was not established with an age and BMI (Table 3). After adjustment for age, weight, and BMI, based on the findings’ analysis for HBA1c between control and football players, it was noticed that HBA1c was significantly lower among the football players compared to control group (*p* = 0.001) (Table 2). 

## 4. Discussion 

This is the first study added in the literature to investigate the prevalence of prediabetes and T2DM among football players compared to population based non-elite athlete control subjects. In this study, a significant decreased was found in the prevalence of prediabetes and T2DM in football players compared to control subjects. The findings were interesting from the perspective that the prevalence of T2DM was significantly low in football players in a nation where T2DM is highest across the globe [16]. 

Meo et al. [17] reported that the prevalence of T2DM in Saudi population with age ranges 29–60 was 32.8%. In another study, Meo et al. [16] identified that in the Arab world nations, highest prevalence of T2DM was in Saudi Arabia (31.6%), followed by Kuwait 25.4%, Bahrain 25.0% and United Arab Emirates 25.0%. As per International Diabetes Federation (IDF) report 2019^3^, worldwide total number of diabetic people with age ranges 20–79 years are 463 million (9.3%)^3^ the prevalence of diabetes in just one country Saudi Arabia is 18.5% [18]. However, as per World Health Organization the prevalence of diabetes in Saudi Arabia is 14.4% [19]. All these findings suggest that T2DM in Saudi Arabia is highest in the region as well as in the world. However, in the present study we found that the prevalence of T2DM in Saudi adult football players was just 1.59% compared to their matched control group (23.54%). These findings suggest that in football players T2DM is astonishingly almost 7 fold less in football players compared to their age, weight, and BMI matched control subjects. 

Football game is packed with a lot of recreational activities, and exercise has been known to positively influence the glucose control [20]. Nieuwoudt et al. [21] determined the changes in beta-cell function after six weeks of high-intensity functional training (HIFT) among 12 sedentary adults with T2DM. After the exercise sessions of 3 days a week, participants showed significant improvements in beta-cell function, while decreasing body fat and preserving lean mass [21]. In another study, Fealy et al. [22] evaluated the effectiveness of their 6-week HIFT intervention for risk factors and reported an increased insulin sensitivity after training. Similarly, Skoradal et al. [1] demonstrated that 16 weeks of football training causes broad-spectrum positive effects on metabolic health profile. It has also been reported that playing football regularly increases insulin sensitivity, positively influencing glycemic control, and potentially providing better tools for the prevention of T2DM [23]. 

Lao et al. [24] evaluated the impact of habitual leisure-time physical activity (LTPA) on T2DM incidence. The authors reported that high levels of LTPA was associated with a lower risk of diabetes. Sarmento et al. [25] demonstrated the benefits of football sport on diseases, including cardiovascular, bone health, and body composition, as sports allied activities increase the insulin sensitivity, and had a positive impact on glycemic control and T2DM. Krustrup et al. [26] describes the health effects of recreational female football players. The authors found that short-term and medium-term recreational football activities have beneficial impact on metabolic health profiles in women. The authors concluded that regular football sport is an effective tool for the prevention of hypertension and T2DM. In another study, Anderson et al. [27] determined the effects of regular football training on glycemic control in men with T2DM. They found that 1-hr football training session, for a 24-week intervention period, caused a greater reduction in plasma glucose. The present study findings shows significant decreased prevalence of prediabetes and T2DM among football players compared to population based non-elite athlete control subjects. 

### 4.1. Possible Mechanism-How Football Reduces Diabetes Mellitus

**Figure 3:**
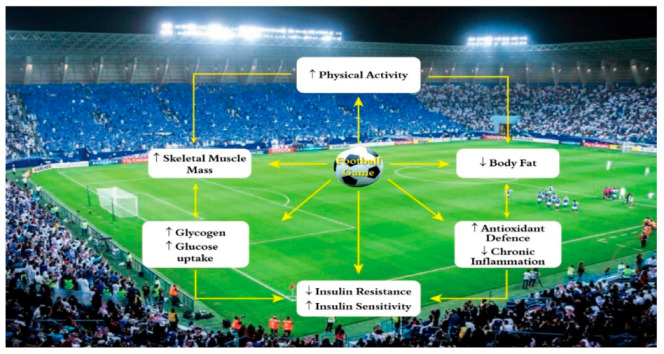
Possible mechanism-how football sport reduces the risk of Type 2 Diabetes Mellitus.

The potential mechanism involved in playing football and decreased insulin resistance and diabetes mellitus is variable. The epidemiological literature acknowledges the fact that football exercise decreases the risk of insulin resistance and ultimately leads to decreased risk of T2DM. Football recreational activities decrease oxidative stress, increase proteins related to mitochondrial biogenesis and improve the antioxidant capabilities along with glucose intake, hence leading to a decrease in T2DM [28] (Figure 3). Moreover, football game improves “insulin sensitivity, enhanced glucose transport into muscle cells [25], and increased production of muscle glycogen to replace the glycogen used during exercise”. These are the possible mechanisms in playing football which lead to the decrease in prevalence of prediabetes and T2DM.

### 4.2. Study Strengths and Limitations 

This is the first study added in the literature to investigate the prevalence of prediabetes and T2DM in football players. The study exclusion criteria were highly standardized and cigarette smokers were excluded. Both groups were matched for age, height, weight, BMI, ethnicity, diet habit, and socio-economic levels to minimize the possible confounding factors. American Diabetes Association diagnosis approach was followed; Glycated Hemoglobin (HbA1c) is a reliable and valid indicator to identify an individual’s long-term mean blood glucose levels and therefore its criteria was employed. This study could therefore be the best reference on the prevalence of prediabetes and T2DM among football players. There are some limitations that we would like to point out: despite trying to recruit large number of football players, we excluded cigarette smokers; age, weight, height, and ethnicity matched criteria was employed, hence we excluded large number of participants and finally included 378 football players and 378 control subjects. Moreover, due to cultural limitations we only included the male gender. 

## 5. Conclusions

Football recreational activities significantly decrease the prevalence of prediabetes and T2DM in football players compared to matched control subjects. The decreased prevalence was associated with the duration of football activities. The study findings have significant public health implications, as findings support the extension of diabetes intervention efforts. Health officials should establish more sport facilities, mainly football grounds, for the public to provide better sports facilities which will help in minimizing the incidence of prediabetes and T2DM. Future prospective studies with large sample sizes are needed to further confirm these findings to get to better conclusions. 

## Figures and Tables

**Table 1:** Sociodemographic and clinical characteristics of football players and matched control subjects (*n* = 756).

Parameters	Football Players (*n* = 378)	Control Group (*n* = 378)	*p*-Value
Age (years)	31.80 ± 5.46	32.32 ± 4.37	0.148
Height (m)	1.72 ± 0.07	1.69 ± 0.07	0.088
Weight (kg)	77.81 ± 6.88	76.85 ± 6.99	0.060
BMI (kg/m^2^)	26.40 ± 2.08	26.66 ± 1.87	0.068

Values are expressed in Mean ± SD.

**Table 2:** Comparison of prevalence of prediabetes and Type 2 Diabetes Mellitus between football players and matched control subjects (*n* = 756).

Parameters	Football Players (*n* = 378)	Control Group (*n* = 378)	*p*-Value
Non-diabetic (HbA1c < 5.7%)	342 (90.48%)	218 (57.67%)	0.001
Prediabetic (HbA1c 5.7–6.4%)	30 (7.96%)	71 (18.78%)	0.001
Diabetic (HbA1c > 6.4%)	6 (1.59%)	89 (23.54%)	0.001

Values are expressed in number and percent. HbA1c values are classified as per American Diabetes Association Guidelines [14].

**Table 3:** Correlations between level of HbA1c and age, Body Mass Index (BMI), and duration of playing football.

Parameters	HBA1C	
	Pearson Correlation Coefficient	*p*-Value
Age (years)	0.026	0.479
BMI (kg/m^2^)	−0.008	0.817
Duration (months)	−0.278 **	<0.001

** Correlation is significant at the 0.01 level (2-tailed).

## Data Availability

Data may be provided on reasonable request to corresponding author.

## References

[1] Skoradal M.-B., Weihe P., Patursson P., Mortensen J., Connolly L., Krustrup P., Mohr M. (2018). Football training improves metabolic and cardiovascular health status in 55- to 70-year-old women and men with prediabetes. Scand. J. Med. Sci. Sports.

[2] Meo S.A. (2009). Diabetes mellitus: Health and wealth threat. Int. J. Diabetes Mellit..

[3] Internatinal Diabetes Fedration Diabetes Atlas. http://www.diabetesatlas.org/key-messages.html.

[4] Wang Q., Zhang X., Fang L., Guan Q., Guan L., Li Q. (2018). Prevalence, awareness, treatment and control of diabetes mellitus among middle-aged and elderly people in a rural Chinese population: A cross-sectional study. PLoS ONE.

[5] World Health Organization Protecting Workers’ Health. https://www.who.int/news-room/fact-sheets/detail/protecting-workers%27-health.

[6] Lee I.-M., Shiroma E.J., Lobelo F., Puska P., Blair S.N., Katzmarzyk P.T. (2012). Effect of physical inactivity on major non-communicable diseases worldwide: An analysis of burden of disease and life expectancy. Lancet.

[7] Faude O., Kerper O., Multhaupt M., Winter C., Beziel K., Junge A., Meyer T. (2010). Football to tackle overweight in children. Scand. J. Med. Sci. Sports.

[8] Krustrup P., Krustrup B.R. (2018). Football is medicine: It is time for patients to play!. Br. J. Sports Med..

[9] Oja P., Titze S., Kokko S., Kujala U.M., Heinonen A., Kelly P., Koski P., Foster C. (2015). Health benefits of different sport disciplines for adults: Systematic review of observational and intervention studies with meta-analysis. Br. J. Sports Med..

[10] Meo S.A., Almutairi F.J., Alasbali M.M., Alqahtani T.B., Almutairi S.S., Albuhayjan R.A., Al Rouq F., Ahmed N. (2018). Men’s Health in Industries: Plastic Plant Pollution and Prevalence of Pre-diabetes and Type 2 Diabetes Mellitus. Am. J. Men’s Health.

[11] Meo S.A., Bin Muneif Y.A., BenOmran N.A., Alsadhan M.A., Hashem R.F., Alobaisi A.S. (2020). Prevalence of Prediabetes and Type 2 Diabetes Mellitus among cement industry workers. Pak. J. Med. Sci..

[12] Kim J.-H., Noh J., Choi J.W., Park E.-C. (2017). Association of Education and Smoking Status on Risk of Diabetes Mellitus: A Population-Based Nationwide Cross-Sectional Study. Int. J. Environ. Res. Public Health.

[13] Majbauddin A., Tanimura C., Aoto H., Otani S., Parrenas M.C.E., Kobayashi N., Morita T., Inoue K., Masumoto T., Kurozawa Y. (2019). Association between dental caries indicators and serum glycated hemoglobin-levels among patients with type 2 diabetes mellitus. J. Oral Sci..

[14] American Diabetes Association (2018). Classification and diagnosis of diabetes: Standards of medical care in diabetes. Diabetes Care.

[15] Sherwani S.I., Khan H.A., Ekhzaimy A., Masood A., Sakharkar M.K. (2016). Significance of HbA1c Test in Diagnosis and Prognosis of Diabetic Patients. Biomark. Insights.

[16] Meo S.A., Usmani A.M., Qalbani E. (2017). Prevalence of type 2 diabetes in the Arab world: Impact of GDP and energy consumption. Eur. Rev. Med. Pharmacol. Sci..

[17] Meo S.A. (2016). Prevalence and future prediction of type 2 diabetes mellitus in the Kingdom of Saudi Arabia: A systematic review of published studies. J. Pak. Med. Assoc..

[18] International Diabetes Fedration (IDF). https://idf.org/our-network/regions-members/middle-east-and-north-africa/members/46-saudi-arabia.html.

[19] World Health Organization. Saudi Arabia. https://www.who.int/diabetes/country-profiles/sau_en.pdf.

[20] Feito Y., Patel P., Redondo A.S., Heinrich K.M. (2019). Effects of Eight Weeks of High Intensity Functional Training on Glucose Control and Body Composition among Overweight and Obese Adults. Sports.

[21] Nieuwoudt S., Fealy C.E., Foucher J.A., Scelsi A.R., Malin S.K., Pagadala M., Rocco M., Burguera B., Kirwan J.P. (2017). Functional high-intensity training improves pancreatic beta-cell function in adults with type 2 diabetes. Am. J. Physiol. Endocrinol..

[22] Fealy C.E., Nieuwoudt S., Foucher J.A., Scelsi A.R., Malin S.K., Pagadala M., Cruz L.A., Li M., Rocco M., Burguera B. (2018). Functional high-intensity exercise training ameliorates insulin resistance and cardiometabolic risk factors in type 2 diabetes. Exp. Physiol..

[23] De Sousa M.V., Fukui R., Krustrup P., Pereira R.M.R., Silva P.R.S., Rodrigues A.C., De Andrade J.L., Hernandez A.J., Da Silva M.E.R. (2014). Positive effects of football on fitness, lipid profile, and insulin resistance in Brazilian patients with type 2 diabetes. Scand. J. Med. Sci. Sports.

[24] Lao X.Q., Deng H.-B., Liu X., Chan T.-C., Zhang Z., Chang L.-Y., Yeoh E.-K., Tam T., Wong M.C.S., Thomas G.N. (2018). Increased leisure-time physical activity associated with lower onset of diabetes in 44 828 adults with impaired fasting glucose: A population-based prospective cohort study. Br. J. Sports Med..

[25] Sarmento H., Clemente F.M., Marques A., Milanović Z., Harper L.D., Figueiredo A. (2020). Recreational football is medicine against non-communicable diseases: A systematic review. Scand. J. Med. Sci. Sports.

[26] Krustrup P., Helge E.W., Hansen T.W., Aagaard P., Hagman M., Randers M.B., De Sousa M., Mohr M. (2018). Effects of recreational football on women’s fitness and health: Adaptations and mechanisms. Graefe’s Arch. Clin. Exp. Ophthalmol..

[27] Andersen T.R., Schmidt J.F., Thomassen M., Hornstrup T., Frandsen U., Randers M.B., Hansen P.R., Krustrup P., Bangsbo J. (2014). A preliminary study: Effects of football training on glucose control, body composition, and performance in men with type 2 diabetes. Scand. J. Med. Sci. Sports.

[28] Busquets-Cortés C., Capó X., Martorell M., Tur J.A., Sureda A., Pons A. (2017). Training and acute exercise modulates mitochondrial dynamics in football players’ blood mononuclear cells. Graefe’s Arch. Clin. Exp. Ophthalmol..

